# Risk factors for incidence and persistence of disability in chronic major depression and alcohol use disorders: Longitudinal analyses of a population-based study

**DOI:** 10.1186/s12955-014-0186-0

**Published:** 2014-12-17

**Authors:** María Cabello, Francisco Félix Caballero, Somnath Chatterji, Alarcos Cieza, José Luis Ayuso-Mateos

**Affiliations:** Instituto de Salud Carlos III, Centro de Investigación Biomédica en Red de Salud Mental (CIBERSAM), Madrid, Spain; Department of Psychiatry, Universidad Autónoma de Madrid, Madrid, Spain; Instituto de Investigación Sanitaria Princesa (IP), La Princesa University Hospital, Madrid, Spain; Department of Health Statistics and Information Systems, World Health Organization, Geneva, Switzerland; Disability and Rehabilitation Unit Coordinator, World Health Organization, Geneva, Switzerland

**Keywords:** Major depression, Alcohol use disorders, Disability

## Abstract

**Background:**

Major depression and alcohol use disorders are risk factors for incidence of disability. However, it is still unclear whether a chronic course of these health conditions is also prospectively associated with incidence of disability. The aim of the present study was, first, to confirm whether chronic major depression (MD) and alcohol use disorders (AUD) are, respectively, risk factors for persistence and incidence of disability in the general population; and then to analyze the role of help-seeking behavior in the course of disability among respondents with chronic MD and chronic AUD.

**Method:**

Data from two assessments in the National Epidemiologic Survey on Alcohol and Related Conditions were analyzed. Disability was measured by eight domains of the Short Form 12 Health Survey version 2 (SF-12). Generalized estimating equations and logistic regression models were run to estimate risk factors for persistence and incidence of disability, respectively.

**Results:**

Analyses conducted on data from the US general population showed that chronic MD was the strongest risk factor for incidence and persistence of disability in the social functioning, emotional role and mental health domains. Chronic AUD were risk factors for incidence and persistence of disability in the vitality, social functioning, and emotional role domains. Within the group of chronic MD, physical comorbidity and help-seeking were associated with persistent disability in most of the SF-12 domains. Help-seeking behavior was also associated with incidence of problems in the mental health domain for the depression group. Regarding the AUD group, comorbidity with physical health problems was a strong risk factor for persistence of disability in all SF-12 domains. Help-seeking behavior was not related to either persistence or incidence of disability in the chronic alcohol group.

**Conclusions:**

Chronic MD and chronic AUD are independent risk factors for persistence and incidence of disability in the US general population. People with chronic MD seek help for their problems when they experience persistent disability, whereas people with chronic AUD might not seek any help even if they are suffering from persistent disability.

## Background

Major depression (MD) and alcohol use disorders (AUD) are both widely recognized as chronic conditions: people with these health conditions suffer from frequent relapses [1,2], and approximately 10-30% of people with these disorders experience a long-lasting course, of more than 24 months [2-5].

Depressive disorders and AUD are also the most important contributors to the burden of mental disorders, as measured in disability-adjusted life years (DALYs), in the United States and Europe [6,7]. The study of the clinical course for these health conditions should also include functioning measures, since people generally consider improved day-to-day functioning as a relevant part of their recovery process [8]. Moreover, problems in functioning are strong predictors for subsequent relapses in these health conditions [9,10].

The literature generally agrees that these health conditions are associated with incidence of disability [11-13]. Remission from symptoms is associated with functional improvements [14]. Increasing evidence has been also reported regarding functional improvements as a result of different treatments [15-18]. However, few studies have analyzed disability trajectories during the chronic course of MD and AUD. In the case of chronic depression, some studies have found that longer duration is associated with higher disability [19,20]. Other studies have suggested that higher disability is a function of greater severity of symptoms and comorbidity, rather than longer duration [21]. However, none of these studies have jointly examined whether chronic MD is a risk factor for both incidence of disability and persistence of disability in the general population.

Regarding chronic AUD, one study has analyzed prospectively the incidence of disability in different chronic groups of AUD [11]. Incidence of disability was found to be higher in people who moved from alcohol abuse to alcohol dependence. No changes in disability were associated with persistent alcohol abuse and persistent alcohol dependence. However, this study did not analyze whether the level of disability experienced by persistent alcohol dependence and persistent alcohol abuse groups was different to the disability experienced by the general population. This study did not analyze either other variables associated with changes in disability scores for the different groups of AUD.

Furthermore, the role of help-seeking behavior in the course of disability has rarely been studied. Generally, help-seeking behavior has been selected as a study outcome. For instance, one study reported that higher disability was an associated factor for help-seeking [22]. Similarly, help-seeking behavior has been associated with better clinical outcomes [23]. Only a single study, to our knowledge, has analyzed the role of use of health services in disability [24]; however, it included both chronic and non-chronic MD patients.

Therefore, the present study aimed to 1) verify whether chronic AUD and chronic MD are risk factors for incidence and persistence of disability in the general population; and 2) specify whether, after controlling for some confounders, help-seeking behavior hinders from incidence of disability in chronic MD and chronic AUD.

## Methods

### Sample

The present study’s sample combined data collected in Wave 1 and Wave 2 of the National Epidemiologic Survey on Alcohol and Related Conditions (NESARC). The NESARC is a representative sample of the non-institutionalized U.S. population 18 years of age and older. Wave 1 (*n* = 43 093, response rate = 81.0%) was collected in 2001–2002, Wave 2 was collected in 2004–2005 (*n* = 34 653, response rate = 86.7%). All the study procedures were in compliance with the Helsinki Declaration guidelines. The NESARC protocol received full ethical approval from the US Census Bureau and the US Office of Management and Budget [25]. All study participants signed a consent form before enrollment. Diagnoses of mental disorders were determined according to DSM-IV, by means of the Alcohol Use Disorders and Associated Disabilities Interview Schedule (AUDADIS) [26]. AUDADIS collects information on both lifetime and 12-month diagnoses. The reliability and consistency of the AUDADIS have been shown to be adequate [27].

Different time periods of diagnosis are considered in NESARC. Diagnoses in Wave 1 are aimed at detecting lifetime prevalence (distinguishing the past year and the period prior to the past year). On the other hand, Wave 2 diagnoses focus on the period since the Wave 1 interview (distinguishing the past year from the period since the last interview, but prior to the past year) [28].

In accordance with the different aims of the present study, three groups of respondents were considered: 1) All NESARC respondents participating in Wave 1 and Wave 2 (*n* = 34 653); 2) respondents with chronic AUD (*n* = 1087); and 3) respondents with chronic MD (*n* = 272). The chronic AUD group was defined as respondents with 12 months prevalence of abuse of or dependence of alcohol in both Wave 1 and Wave 2 interviews, and also in the period elapsed before the Wave 1 interview and before Wave 2 (two years of follow–up). Similarly, the chronic MD group was defined as respondents who persistently met DSM-IV criteria for MD in the 12 months prior to Wave 1, the 12 months prior to Wave 2, and also in the period between these two interviews.

### Variables

#### Disability

NESARC assessed disability by means of the Short Form 12 Health Survey version 2 (SF-12v2) [29]. SF-12v2 collects information from eight functioning domains: social functioning, general health, vitality, physical functioning, physical role, mental health, emotional role, and body pain; it has shown adequate statistical properties in the general population [30]. SF-12v2 scores on NESARC included norm-based disability scores in a measure ranging from 0 to 100, with lower scores indicating more severe disability. In the present study, the presence of disability was defined as a score lower than or equal to the 25th percentile for each SF-12 domain. This cut-off point has been previously considered as adequate to define groups with disability [31].

Therefore, persistence of disability was defined as the presence of disability in both time assessments. Incidence of disability was calculated selecting respondents who ranked > 25th percentile at wave 1, but then scored ≤ 25th percentile at wave 2.

#### Socio-demographics

Age (in years), gender (male/female), being unemployed (yes/no) (including as unemployed both those looking and not looking for a job), family annual income (21 ordered levels, ranging from $0 to more than $200,000), and educational attainment (14 ordered levels, ranging from no formal schooling to master’s degree or higher). Family annual income and educational attainment were treated as continuous variables. These variables have been independently related to disability by previous literature [32].

#### Physical health conditions

Self-reported diagnosis of atherosclerosis, hypertension, angina, chest pain, heart attack, myocardial infarction, other heart diseases, gastritis, arthritis, and cirrhosis of the liver or other liver diseases were considered at the two time assessments. Physical health condition was defined as suffering from at least one of these diseases for each time assessment. These physical health conditions have been included because they are relatively frequent in people with mental disorders and their comorbidity is associated with a great burden [33].

#### Mental health disorders

12 months’ prevalence of Axis I disorders were collected by means of AUDADIS interview at both time assessments. Axis I disorders considered were anxiety disorders, other mood disorders such as dysthymia, mania or hypomania episodes, and self-reported diagnosis of schizophrenia. Consequently, this variable was defined as experiencing at least one of these diseases. Two different variables of mental disorders were created for each time assessment. MD was considered in this variable for the analyses including the chronic AUD group. Similarly, for specific depression analyses, AUD were included into this variable to consider their impact on the chronically depressed.

#### Severity of symptoms

Severity of MD and AUD was measured counting the number of criteria met for each of the diagnoses respectively. AUDADIS items for collecting diagnoses of MD were grouped into 10 sets of symptoms derived from ICD-10 criteria [34], as follows: low mood or sadness; loss of interest; tired/energy problems; weight- or appetite-related problems; sleep problems; problems of self-esteem or feelings of worthlessness; feelings of guilt or inadequacy; thought or concentration problems; suicidal ideation or attempts; and agitation or retardation problems. A summary score of these groups of symptoms, ranging from five (minimum criteria for establishing major depression, according to DSM-IV) to 10 was obtained for the two time assessments. Previous studies including NESARC data have already used a summary score of the number of depressive symptoms as an approach for measuring severity of depression [35]. Similarly, AUDADIS items for diagnosis of AUD were grouped into 10 sets of symptoms (yes/no) as follows: taking the substance in larger amounts or for longer than the you meant to; wanting to cut down or stop using the substance but not managing to; spending a lot of time getting, using, or recovering from use of the substance; not managing to do what you should at work, home or school, because of substance use; continuing to use, even when it causes problems in relationships; giving up important social, occupational or recreational activities because of substance use; using substances again and again, even when it puts the you in danger; continuing to use, even when the you know you have a physical or psychological problem that could have been caused or made worse by the substance; needing more of the substance to get the effect you want (tolerance); and development of withdrawal symptoms, which can be relieved by taking more of the substance. Summary scores ranging from 1 to 10 were calculated in order to measure severity of AUD at the two time assessments. This severity score has been also previously used [36] and is a reliable measure for measuring severity of AUD [37].

#### Health care-seeking behavior

This variable was defined as visiting at least one health service for the last 12 months. However, different types of health services were considered for each health condition. Help-seeking in AUD was defined as visiting at least one of the following services: Alcoholics Anonymous; family services or other social services; alcohol/drug detoxification ward/clinic; inpatient ward of psychiatric/general hospital; community mental health program; outpatient clinic; alcohol/drug rehabilitation program; halfway house/therapeutic community; crisis center; Employee Assistance Program (EAP); private health professional (physician, psychologist, social worker or psychiatrist); emergency room; and seeking a clergyman, rabbi or priest or some other agency because of alcohol use problems. Additionally, help-seeking behavior was defined in MD as scoring “yes” on at least some of these health services: visiting a therapist/physician, staying at emergency room or at overnight hospital, or “doctor prescribed medicine/drug to improve my mood”.

### Statistical analyses

Firstly, prevalence estimates of persistence and incidence of disability were estimated for the three different populations: general population, chronic MD, and chronic AUD. Standard errors were estimated using the Taylor series linearization method [38] to adjust for the complex sample design.

Secondly, analyses including persistent disability as the outcome were separately carried out for the global sample, and the chronic MD and chronic AUD groups. A methodology based on Generalized Estimating Equations (GEE) was employed. GEE analysis were conducted for panel data, clustered by ID, and run using a population-averaged model [39]. A logit link function and an exchangeable correlation structure were used to account for the binary nature of outcome variables and the correlation between observations in the same individual. The robust variance estimator was used to account for the within-subject correlation. Chronic MD and chronic AUD groups were considered as predictor variables in the GEE analysis for the general population. Additionally, physical health conditions, mental health conditions (considering Axis I comorbidity), educational level, family income level, unemployment, gender and age were included in the GEE model as covariates to control their potential confounder effect on the persistence of disability. These aforementioned variables together with severity of symptoms and help-seeking behavior were considered as predictors for the analyses conducted with chronic MD and AUD groups.

Three different multiple logistic regression analyses were run to estimate predictors of incidence of disability at Wave 2 for the general population, chronic MD and chronic AUD groups. Only a subgroup of the population with no disability at Wave 1 (i.e., scoring higher than the 25th percentile) was considered for these analyses. Particularly for general population analyses of incidence, chronic MD and chronic AUD were respectively defined as the presence of MD and AUD in the 12 month prior to Wave 1 and in the period elapsed from Wave 1 to 12 months prior to Wave 2. This definition assured that MD and AUD episodes occurred before incidence of disability in Wave 2. The rest of demographic variables, physical health conditions and mental health disorders (Axis I disorders) collected in Wave 1 were included as predictors to control their confounder effect on incidence of disability in Wave 2. The previously mentioned demographic and clinical variables, together with severity of symptoms in Wave 1 and help-seeking behavior in Wave 1, were included as predictors in the specific analyses of incidence for the groups of chronic MD and AUD. Odds-ratios (OR) and confidence intervals (CI) at the 95% confidence level were calculated in both GEE models and the logistic regression analyses.

All statistical analyses were weighted using Wave 2 weights and were conducted for each SF-12 domain. NESARC data were weighted in both Wave 1 and 2. In both waves, weights were adjusted to match the civilian, non-institutionalized population of the United States with respect to distribution by age, sex, race, ethnicity and region, based on the 2000 Decennial Census. Moreover, Wave 2 weights were also adjusted for non-response relative to Wave 1 lifetime substance use and other psychiatric disorders. Deeper details of weighting procedures are described elsewhere [28]. All analyses were conducted using Stata version 11 [40].

## Results

### Prevalence estimates of disability

Prevalence estimates for persistence and incidence of disability are shown in Table 1. Persistence rates were generally higher than incidence rates for the general population and for the chronic MD and AUD groups. People with chronic MD experienced higher persistence of disability than the general population for all the SF-12 domains. However, only estimates of persistent problems in mental health-related domains were significantly higher in the chronic AUD group than in the general population.

**Table 1:** **Prevalence estimates (95% CI) of persistence of disability and incidence of disability in the general population, population with chronic major depression, and population with chronic alcohol use disorders**

	**General population (** ***n*** **= 34 653)**	**Chronic major depression (** ***n*** **= 272)**	**Chronic alcohol use disorders (** ***n*** **=1087)**
***Persistence of disability***			
Physical functioning	16.69% (16.22 to 17.16)	**28.33% (21.67 to 34.99)**	**10.15% (7.88 to 12.43)**
Physical role	14.80% (14.35 to 15.24)	**30.70% (23.96 to 37.44)**	**10.39% (8.09 to 12.69)**
Bodily pain	20.86% (20.34 to 21.38)	**43.42% (36.13 to 50.70)**	21.07% (18.05 to 24.09)
General health	27.19% (26.62 to 27.76)	**45.91% (38.65 to 53.17)**	27.27% (24.03 to 30.52)
Vitality	24.96% (24.40 to 25.52)	**63.82% (56.96 to 70.68)**	26.16% (23.01 to 29.32)
Social functioning	11.71% (11.31 to 12.12)	**47.92% (40.69 to 55.16)**	**17.33% (14.51 to 20.14)**
Emotional role	14.06% (13.62 to 14.50)	**49.24% (41.97 to 56.50)**	**19.30% (16.37 to 22.24)**
Mental health	16.27% (15.80 to 16.74)	**56.58% (49.36 to 63.80)**	**22.92% (19.88 to 25.96)**
***Incidence of disability***			
Physical functioning	11.80% (11.39 to 12.21)	**18.97% (13.16 to 24.77)**	10.99% (8.64 to 13.34)
Physical role	10.53% (10.13 to 10.93)	9.76% (6.03 to 13.50)	11.11% (8.98 to 13.25)
Bodily pain	15.81% (15.33 to 16.29)	18.63% (12.87 to 24.38)	15.54% (12.99 to 18.10)
General health	13.99% (13.54 to 14.44)	11.99% (7.23 to 16.76)	13.61% (11.15 to 16.07)
Vitality	19.03% (18.52 to 19.53)	16.46% (11.40 to 21.53)	19.78% (16.88 to 22.67)
Social functioning	14.15% (13.71 to 14.60)	19.56% (13.48 to 25.63)	16.79% (14.12 to 19.46)
Emotional role	16.08% (15.60 to 16.55)	16.32% (10.58 to 22.05)	19.18% (16.32 to 22.04)
Mental health	15.91% (15.43 to 16.38)	18.73% (12.80 to 24.66)	16.96% (14.25 to 19.66)

Note:** In bold**, prevalence estimates for chronic major depression and chronic alcohol use disorder whose 95% CI do not overlap with 95% CI for the prevalence estimate in the general population.

In the general population and in the chronic AUD group, the highest prevalence of persistent disability was found for the general health domain (27.19%, 95% CI = 26.62 to 27.76, in the general population; 27.27%, 95% CI = 24.03 to 30.52 in the chronic AUD group). In contrast, persistent disability was highly prevalent for the vitality domain in the group of chronic MD (63.82%, 95% CI = 56.96 to 70.68). Regarding the incidence of disability, the prevalence estimates ranged from 10.53% (physical role) to 19.03% (vitality) in the general population. The highest incidence rates were found for social functioning (19.56%, 95% CI = 13.48 to 25.63) and vitality (19.78%, 95% CI = 16.88 to 22.67) in the chronic MD and chronic AUD groups, respectively.

### Risk Factors for Disability in the General Population

Risk factors for disability in the general population are shown in Table 2. After controlling for socio-demographics and other health conditions, chronic MD was the strongest risk factor for persistence of disability in the emotional role, social functioning and mental health domains in the general population. The presence of physical health conditions was the main predictor of persistent disability in the physical functioning, physical role, bodily pain, general health, and vitality domains. Chronic AUD also had an impact on persistence of disability, except for in the physical-health related domains. Similar results were found in the analyses to determine risk factors for incidence of disability in the general population (Table 2). Chronic MD and the presence of physical health problems were the strongest risk factors for incidence of disability in physical functioning and bodily pain, while the presence of physical health problems was the strongest risk factor for incidence of disability in the remaining physical-health related domains. Moreover, chronic MD was the strongest risk factor for incidence of disability in the social functioning, vitality, emotional role and mental health domains. Chronic AUD was a risk factor for incidence of disability in the vitality, social functioning and emotional role domains. Lower family income, lower educational attainment and unemployment were related to persistence and incidence of disability in the general population.

**Table 2:** **Odds ratio (95% CI) associated with predictors for persistence of disability and incidence of disability in the general population**

	**Physical functioning**	**Physical role**	**Bodily pain**	**General health**	**Vitality**	**Social functioning**	**Emotional role**	**Mental mealth**
***Persistence of disability***								
Educational attainment	0.92***(0.91 to 0.93)	0.93***(0.92 to 0.94)	0.95***(0.94 to 0.96)	0.85***(0.84 to 0.86)	0.97***(0.96 to 0.98)	0.98***(0.97 to 0.99)	0.97***(0.96 to 0.98)	0.97***(0.96 to 0.98)
Family income	0.93***(0.92 to 0.94)	0.93***(0.92 to 0.94)	0.96***(0.95 to 0.97)	0.94***(0.93 to 0.95)	0.96***(0.95 to 0.97)	0.94***(0.93 to 0.95)	0.94***(0.93 to 0.95)	0.96***(0.95 to 0.97)
Unemployment	1.38***(1.24 to 1.54)	1.43***(1.28 to 1.58)	1.13*(1.02 to 1.25)	1.43***(1.30 to 1.57)	1.06(0.97 to 1.17)	1.50***(1.36 to 1.66)	1.54***(1.39 to 1.70)	1.48***(1.34 to 1.63)
Physical health conditions	3.25***(3.08 to 3.43)	2.99***(2.83 to 3.15)	3.40***(3.23 to 3.58)	2.47 ***(2.35 to 2.60)	2.19***(2.08 to 2.30)	2.30***(2.18 to 2.43)	1.95***(1.86 to 2.05)	1.73***(1.64 to 1.82)
Mental health conditions	1.77***(1.67 to 1.88)	1.64***(1.55 to 1.74)	1.78***(1.68 to 1.88)	1.55***(1.47 to 1.63)	1.87***(1.78 to 1.97)	2.45***(2.31 to 2.59)	2.40***(2.27 to 2.54)	2.26***(2.14 to 2.38)
Major depression	1.49***(1.34 to 1.65)	1.50***(1.35 to 1.67)	1.58***(1.43 to 1.74)	1.50***(1.36 to 1.64)	2.13***(1.93 to 2.35)	2.74***(2.48 to 3.02)	2.99***(2.71 to 3.30)	3.30***(2.99 to 3.63)
Alcohol use disorders	0.96(0.86 to 1.06)	0.99(0.90 to 1.09)	1.16***(1.07 to 1.26)	1.07(0.98 to 1.16)	1.09*(1.01 to 1.18)	1.35***(1.23 to 1.47)	1.31***(1.21 to 1.43)	1.17***(1.08 to 1.26)
***Incidence of disability***								
Educational attainment	0.94***(0.92 to 0.96)	0.94***(0.92 to 0.96)	0.94***(0.93 to 0.96)	0.87***(0.85 to 0.88)	0.97***(0.95 to 0.98)	0.96***(0.95 to 0.98)	0.95***(0.93 to 0.97)	0.95***(0.93 to 0.96)
Family income	0.96***(0.95 to 0.97)	0.96***(0.95 to 0.97)	0.99(0.98 to 1.01)	0.96***(0.95 to 0.97)	0.98***(0.97 to 0.99)	0.96***(0.95 to 0.97)	0.96***(0.95 to 0.97)	0.98***(0.97 to 0.99)
Unemployment	1.71***(1.39 to 2.10)	1.50***(1.22 to 1.85)	1.47***(1.23 to 1.76)	1.31**(1.07 to 1.61)	1.18(0.98 to 1.43)	1.60***(1.33 to 1.93)	1.21(0.99 to 1.48)	1.41***(1.17 to 1.71)
Physical health conditions	1.96***(1.76 to 2.18)	1.67***(1.49 to 1.88)	1.89***(1.70 to 2.11)	1.58***(1.41 to 1.78)	1.48***(1.34 to 1.64)	1.54***(1.39 to 1.70)	1.52***(1.38 to 1.67)	1.25***(1.13 to 1.38)
Mental health conditions	1.33***(1.18 to 1.51)	1.37***(1.21 to 1.56)	1.37***(1.22 to 1.54)	1.04(0.91 to 1.17)	1.27***(1.14 to 1.43)	1.47***(1.31 to 1.64)	1.46***(1.30 to 1.64)	1.30***(1.15 to 1.46)
Major depression	2.06***(1.49 to 2.84)	1.29(0.90 to 1.86)	2.02***(1.41 to 2.88)	1.44*(1.01 to 2.10)	3.05***(2.00 to 4.67)	4.44***(3.02 to 6.52)	3.66***(2.31 to 5.82)	4.82***(3.03 to 7.68)
Alcohol use disorders	1.19(0.94 to 1.50)	1.22(0.98 to 1.51)	1.19(0.98 to 1.45)	1.03(0.83 to 1.27)	1.28*(1.05 to 1.54)	1.46***(1.20 to 1.78)	1.59**(1.31 to 1.92)	1.19(0.97 to 1.46)

Note: *p < 0.05; **p < 0.01; ***p < 0.001; Analyses were controlled by age and sex. In the models predicting incidence of disability, participants with severe disability in Wave 1, in the domain assessed, were excluded from the analysis corresponding to that domain.

### Risk factors for disability in people with chronic MD

Table 3 shows the risk factors for persistence and incidence of disability in respondents with chronic MD. Physical comorbidity was the strongest risk factor for persistence of disability in the physical functioning, physical role, bodily pain, general health, and vitality domains for respondents with this health condition. MD was also associated with social functioning and emotional role. Help-seeking behavior was significantly associated with persistent problems in the physical functioning, physical role, general health and emotional role domains. Severity of depressive symptoms was a risk factor for persistence in the general health, social functioning, emotional role and mental health domains. Low family income was related with persistence of disability for all SF-12 domains except for bodily pain and physical role, while unemployment and low educational attainment were related with persistence of disability in some of the physical health-related domains.

**Table 3:** **Odds ratio (95% CI) associated with predictors for persistence of disability and incidence of disability, in the population with chronic major depression**

	**Physical functioning**	**Physical role**	**Bodily pain**	**General health**	**Vitality**	**Social functioning**	**Emotional role**	**Mental health**
***Persistence of disability***								
Educational attainment	0.88*(0.77 to 0.98)	0.96(0.85 to 1.08)	0.89(0.79 to 1.01)	0.82**(0.72 to 0.93)	1.00(0.88 to 1.13)	1.05(0.93 to 1.17)	0.99(0.89 to 1.11)	0.93(0.82 to 1.06)
Family income	0.92**(0.87 to 0.98)	0.95(0.90 to 1.01)	0.97(0.92 to 1.03)	0.94*(0.89 to 0.99)	0.92**(0.87 to 0.98)	0.91**(0.86 to 0.96)	0.92**(0.87 to 0.98)	0.90**(0.84 to 0.95)
Unemployment	1.39(0.68 to 2.82)	1.96*(1.04 to 3.68)	1.42(0.66 to 3.05)	1.33(0.79 to 2.23)	2.57*(1.06 to 6.24)	1.48(0.64 to 3.40)	1.02(0.44 to 2.34)	1.68(0.57 to 4.93)
Physical health conditions	4.00***(2.29 to 6.97)	5.11***(2.96 to 8.80)	4.87***(2.71 to 8.73)	2.25**(1.35 to 3.75)	3.34***(1.75 to 6.38)	1.77*(1.06 to 2.98)	2.29*(1.20 to 4.37)	1.58(0.86 to 2.88)
Mental health conditions	1.10(0.69 to 1.75)	0.84(0.53 to 1.33)	1.12(0.74 to 1.69)	1.20(0.84 to 1.72)	0.63(0.36 to 1.08)	1.19(0.75 to 1.87)	1.17(0.73 to 1.88)	1.04(0.62 to 1.75)
Severity of depression	1.03(0.86 to 1.23)	1.09(0.93 to 1.27)	1.11(0.93 to 1.32)	1.32**(1.12 to 1.55)	1.20(0.99 to 1.46)	1.40***(1.18 to 1.66)	1.28*(1.03 to 1.58)	1.25*(1.03 to 1.52)
Seeking help for major depression	2.01**(1.19 to 3.38)	1.73**(1.16 to 2.58)	1.52(0.96 to 2.42)	1.57*(1.05 to 2.36)	1.71(0.96 to 3.03)	1.43(0.88 to 2.32)	1.80*(1.04 to 3.10)	1.48(0.82 to 2.65)
***Incidence of disability***								
Educational attainment	0.78*(0.64 to 0.96)	0.92(0.73 to 1.17)	0.91(0.73 to 1.14)	0.70*(0.54 to 0.92)	0.83(0.62 to 1.11)	0.82(0.64 to 1.05)	0.78(0.57 to 1.07)	0.65*(0.45 to 0.95)
Family income	0.98(0.89 to 1.07)	0.94(0.84 to 1.06)	0.98(0.88 to 1.10)	1.01(0.87 to 1.16)	1.03(0.89 to 1.19)	0.86*(0.74 to 0.98)	1.08(0.92 to 1.25)	1.03(0.87 to 1.21)
Unemployment	3.98*(1.05 to 15.07)	1.24(0.25 to 6.26)	0.95(0.14 to 6.26)	1.55(0.17 to 14.17)	12.87*(1.15 to 144.69)	0.40(0.08 to 1.98)	0.11(0.01 to 1.58)	0.15(0.01 to 4.42)
Physical health conditions	3.13*(1.01 to 9.78)	0.92(0.14 to 5.99)	2.54(0.58 to 11.10)	4.80*(1.30 to 17.71)	14.66*(1.12 to 192.35)	6.38*(1.42 to 28.67)	4.19(0.68 to 25.93)	1.19(0.26 to 5.50)
Mental health conditions	1.51(0.55 to 4.14)	0.80(0.29 to 2.20)	1.15(0.45 to 2.97)	1.21(0.40 to 3.71)	2.09(0.62 to 7.04)	1.13(0.31 to 4.08)	0.51(0.15 to 1.67)	1.60(0.33 to 7.67)
Severity of depression	0.74(0.50 to 1.09)	0.97(0.62 to 1.51)	0.89(0.63 to 1.27)	0.79(0.54 to 1.15)	1.30(0.80 to 2.10)	1.07(0.63 to 1.80)	1.20(0.84 to 1.73)	0.69(0.43 to 1.12)
Seeking help for major depression	0.60(0.20 to 1.75)	0.73(0.21 to 2.50)	1.54(0.51 to 4.69)	0.98(0.26 to 3.71)	0.58(0.11 to 3.05)	1.73(0.49 to 6.07)	4.85(0.70 to 33.60)	6.81**(1.83 to 25.43)

Note: *p < 0.05; **p < 0.01; ***p < 0.001; Analyses were controlled by age and sex. In the models predicting incidence of disability, participants with severe disability in Wave 1, in the domain assessed, were excluded from the analysis corresponding to that domain.

Regarding incidence of disability, unemployment was the strongest risk factor for the physical functioning (OR = 3.98; 95% CI = 1.05 to 15.07) and vitality (OR = 12.87; 95% CI = 1.15 to 144.69) domains. Comorbidity with physical health conditions was a risk factor for incident disability in physical functioning, general health, vitality and social functioning. Finally, help-seeking behavior was significantly related to incidence of disability in the mental health domain (OR = 6.81; 95% CI = 1.83 to 25.43).

### Risk factors for disability in people with chronic AUD

Finally, the results for the chronic AUD group are shown in Table 4. Regarding persistence of disability, comorbid physical disorders had the highest impact on the physical functioning, physical role, bodily pain, social functioning, vitality and general health domains, while the comorbid mental disorders variable was the strongest risk factor for mental health-related domains. Severity of AUD was associated with persistent disability for all SF-12 domains. Unemployment was strongly related with persistent disability in mental health (OR = 2.10; 95% CI = 1.38 to 3.20). Help-seeking behavior was marginally related with persistence of disability in social functioning (OR = 1.55, 95% CI = 0.99 to 2.45; *p* = 0.058).

**Table 4:** **Odds ratio (95% CI) associated with predictors for persistence of disability and incidence of disability, in the population with chronic alcohol use disorders**

	**Physical functioning**	**Physical role**	**Bodily pain**	**General health**	**Vitality**	**Social functioning**	**Emotional role**	**Mental health**
***Persistence of disability***								
Educational attainment	0.94(0.86 to 1.03)	0.92(0.85 to 1.01)	0.93*(0.87 to 0.99)	0.83***(0.77 to 0.90)	1.04(0.97 to 1.11)	1.05(0.99 to 1.12)	1.05(0.98 to 1.12)	1.02(0.96 to 1.09)
Family income	0.92**(0.90 to 0.95)	0.95*(0.92 to 0.98)	0.95***(0.92 to 0.97)	0.96**(0.94 to 0.98)	0.98(0.95 to 1.01)	0.98(0.96 to 1.01)	0.97**(0.94 to 0.99)	0.99(0.96 to 1.01)
Unemployment	1.71**(1.10 to 2.67)	2.05**(1.28 to 3.29)	1.30(0.87 to 1.96)	1.75*(1.14 to 2.69)	1.01(0.68 to 1.49)	1.53*(1.02 to 2.31)	1.40(0.91 to 2.16)	2.10**(1.38 to 3.20)
Physical health conditions	3.16***(2.27 to 4.41)	2.91***(2.06 to 4.12)	3.06***(2.22 to 4.21)	3.11***(2.28 to 4.23)	1.64**(1.20 to 2.25)	2.05***(1.48 to 2.84)	1.51**(1.08 to 2.10)	1.40*(1.02 to 1.92)
Mental health conditions	1.49***(1.10 to 2.02)	1.74**(1.29 to 2.36)	1.47**(1.14 to 1.90)	1.46**(1.15 to 1.85)	1.59***(1.27 to 2.00)	2.04***(1.57 to 2.66)	2.24***(1.74 to 2.88)	2.09***(1.64 to 2.66)
Severity of alcohol use	1.13**(1.05 to 1.22)	1.13***(1.05 to 1.21)	1.12***(1.06 to 1.18)	1.11***(1.05 to 1.17)	1.14***(1.07 to 1.20)	1.23***(1.16 to 1.31)	1.21***(1.14 to 1.28)	1.17***(1.10 to 1.23)
Seeking help for drinking problems	1.17(0.71 to 1.94)	1.02(0.60 to 1.74)	0.95(0.63 to 1.43)	0.88(0.57 to 1.34)	1.11(0.73 to 1.68)	1.55(0.99 to 2.45)	1.06(0.70 to 1.59)	1.28(0.84 to 1.97)
***Incidence of disability***								
Educational attainment	0.93(0.81 to 1.06)	0.92(0.81 to 1.03)	0.91(0.82 to 1.02)	0.86*(0.76 to 0.98)	0.98(0.88 to 1.09)	1.02(0.91 to 1.14)	1.01(0.91 to 1.12)	0.98(0.87 to 1.09)
Family income	0.94(0.87 to 1.01)	1.03(0.97 to 1.08)	0.96(0.91 to 1.01)	0.98(0.93 to 1.03)	1.00(0.96 to 1.05)	1.00(0.95 to 1.04)	1.02(0.97 to 1.07)	1.00(0.95 to 1.05)
Unemployment	1.95(0.80 to 4.75)	1.91(0.83 to 4.38)	1.46(0.66 to 3.20)	2.48*(1.03 to 5.94)	1.65(0.70 to 3.87)	3.11**(1.47 to 6.58)	1.46(0.67 to 3.19)	1.54(0.65 to 3.67)
Physical health conditions	1.84(0.95 to 3.56)	3.16***(1.71 to 5.86)	1.74(0.93 to 3.27)	1.30(0.60 to 2.83)	1.39(0.77 to 2.51)	1.48(0.83 to 2.64)	1.72(0.94 to 3.12)	0.94(0.51 to 1.72)
Mental health conditions	1.35(0.69 to 2.62)	1.33(0.73 to 2.42)	1.19(0.71 to 1.98)	1.83*(1.04 to 3.19)	1.41(0.84 to 2.37)	1.62(0.99 to 2.64)	1.42(0.82 to 2.46)	1.21(0.68 to 2.15)
Severity of alcohol use	1.13(0.99 to 1.27)	1.07(0.94 to 1.21)	1.01(0.90 to 1.13)	1.02(0.91 to 1.14)	1.01(0.91 to 1.12)	1.05(0.93 to 1.18)	1.24***(1.11 to 1.38)	1.05(0.95 to 1.17)
Seeking help for drinking problems	1.94(0.80 to 4.72)	1.62(0.74 to 3.57)	2.13(0.99 to 4.55)	1.46(0.59 to 3.61)	1.36(0.60 to 3.09)	1.71(0.80 to 3.65)	1.52(0.68 to 3.38)	1.30(0.57 to 2.94)

Note: *p < 0.05; **p < 0.01; ***p < 0.001; Analyses were controlled by age and sex. In the models predicting incidence of disability, participants with severe disability in Wave 1, in the domain assessed, were excluded from the analysis corresponding to that domain.

Regarding incidence of disability, comorbidity with mental disorders was a predictor for developing disability in general health (OR = 1.83; 1.04 to 3.19). Comorbidity with physical disorders was the strongest risk factor for worsening in the physical role domain (OR = 3.16; 95% CI = 1.71 to 5.86). Additionally, severity of AUD was a relevant risk factor for incident disability in emotional role, and was marginally associated with the incidence of disability in physical functioning (OR = 1.13, 95% CI = 0.99 to 1.27; *p* = 0.052). Help-seeking behavior was not significantly associated with incident disability, but some marginally significant trend of relationship could be observed; for example, in bodily pain (OR = 2.13, 95% CI = 0.99 to 4.55; *p* = 0.052). Lower family income was also marginally related with incidence of disability in physical functioning and bodily pain.

## Discussion

The present study is the first to prospectively examine whether chronic course of MD and chronic AUD are risk factors for incidence and persistence of disability in the general population as well as to report specific risk factors for incidence and persistence of disability in these health conditions.

We found that, by and large, prevalence estimates for persistence of disability were higher than for incidence in the general population, as well as among respondents with chronic MD or chronic AUD. This is an expected result, if we consider that NESARC included on average three years between time assessments, and this period may not be a long enough interval to study incidence of disability in a relatively younger community-dwelling population.

Our findings clearly indicate that chronic MD is a risk factor for persistent disability in the general population. This is congruent with previous studies reporting that a long-lasting course of MD is associated with persistent disability [41,42]. People at higher risk of chronic depression may require longer treatments to avoid a persistent course of disability. According to relevant experts [43], key components of these treatments may be longer treatment periods and careful adherence monitoring.

Another important finding of the present study is that even after controlling for other potential confounders, chronic MD was associated with incidence of disability in a variety of domains. This finding is in the line with a previous longitudinal study [19]. However, other studies have reported that recurrence and longer duration of depression were not risk factors for higher disability in MD [21,24,44]. Several methodological differences might explain these differences. Firstly, chronicity was not measured prospectively in these previous studies. Secondly, recurrence and persistence were analyzed separately. In addition, disability scores were compared to patients with non-chronic depression and not to the general population, as done here. All these differences hinder study comparisons. Nevertheless, future longitudinal studies should compare whether individuals with chronic and non-chronic depression present similar risk for persistence and incidence of disability.

Our results also indicate that the presence of chronic AUD is a risk factor for persistence of disability in a wide range of functional areas, except for physical health-related domains. Some studies have previously reported that AUD are associated with mental health-related problems rather than physical ones [45,46]. These results could be explained by the fact that physical problems might be associated with comorbidity and social exclusion factors, particularly frequent in people with AUD [47]. Nevertheless, our findings suggest that chronic AUD might be considered an important risk factor for chronic disability in the general population. Treatments in AUD should be aimed at improving all those functional areas suffering long-lasting impairment, not only focusing on achieving complete long-term abstinence.

On the other hand, chronic AUD were risk factors for incidence of disability in the domains of vitality, social functioning and emotional role. This finding may suggest that disability in chronic AUD could be mostly persistent, and that only specific areas might be at higher risk of worsening over time. However, further studies should verify whether chronic AUD are risk factors for both incidence and persistence of disability over the longer term.

In addition, the present study is the first to analyze a wide range of risk factors for both persistence and incidence of disability in people with chronic MD and AUD. Comorbid physical disorders were strong risk factors for persistence of disability for most of the domains in both MD and AUD. This finding is congruent with the idea that physical comorbidity among mental disorders is highly disabling [33]. Further research is necessary to test tailored mental health interventions for people with comorbid physical disorders [48], since people with physical diseases (particularly some cardiovascular and liver diseases) have been frequently excluded from psychopharmacological trials.

Another important finding was that help-seeking behavior for mood problems was related to persistent disability in chronic MD. This outcome might be explained by the fact that people with MD seek help when they are suffering from more severe disability [49,50]. In contrast, health care -seeking behavior for drinking problems was not significantly related to persistence of disability in people with chronic AUD. This finding might suggest that people with chronic AUD did not seek help in spite of suffering from persistent problems of functioning. More comprehensive treatment approaches, rather than emphasizing immediate abstinence, might be necessary to motivate treatment entry in persons with AUD [51]. As opposed to our hypothesis, help-seeking behavior at Wave 1 did not protect from incidence of disability at Wave 2 in the either group, whether chronic MD or AUD. In addition, help-seeking behavior was identified as a risk factor for incidence of disability at Wave 2, specifically for mental health in the MD group, and marginally for bodily pain in AUD. There may be different reasons that explain why help-seeking behavior did not provide protection from incidence of disability. One is that people with chronic AUD and MD who were seeking help at baseline scored significantly lower in self-perception of health (results available at authors’ request). It has been previously reported in the literature that self-perception of health is longitudinally related to level of functioning [52]; consequently, respondents who had already sought help at baseline could suffer from sub-threshold severe problems, and easily developed incidence of severe disability more often. Another possible explanation could be that help-seeking behavior has nothing to do with receiving high-quality treatment or with treatment compliance, which could be the factors directly related to better functional outcomes [53]. Further studies should confirm how help-seeking behavior is related to subsequent disability.

As expected, higher severity of symptoms was also related to persistence of disability in all of the SF-12 domains in AUD, and for mental-health related domains in the MD group. However, severity was not as strong a risk factor for disability as has been reported in previous studies [44]. In addition, a higher number of symptoms was not a relevant factor for developing new disability for either the AUD or MD groups. This finding could be due to people with comorbid health conditions also experiencing higher severity of symptoms.

Regarding socio-demographic variables, unemployment was a risk factor for persistence and incidence of disability in the general population. Consequently, societies with high rates of unemployment are at higher risk for developing severe and chronic disability. More effective policies should be applied to face the rates of unemployment that the US and some European countries are still suffering, particularly among the younger and less educated populations [54]. Unemployment was also related to persistent disability for a variety of disability domains in both chronic MD and AUD, and with incidence of disability in chronic MD. Previous studies have already shown that people with mental disorders are vulnerable to being unemployed and to losing their jobs [55]. More efforts should be made to ensure employment equity for people with mental problems.

Analyzing other demographic risk factors in the groups of specific health conditions, the current findings also confirm that the burden of chronic MD may be particularly dramatic for people with less resources, since lower family income and a lower education level were also risk factors for persistent disability in chronic MD. Lower income was also marginally related with incidence of disability in some physical health-related domains in chronic AUD. Mental health policymakers should focus their efforts on people with less resources in order to address the inequalities that mental disorders cause. The literature has suggested some actions that can promote mental health, maintaining good health care services and not compromising the care of citizens in times of economic recession [56].

The present study should be also interpreted with the following limitations in mind. Our report has defined incidence of disability by the significant switch from a “normal” level of functioning to an “impaired” level of functioning. Persistence of disability was defined by the maintenance of the same impaired level of functioning. Although our definition of disability based on 25th percentile has been previously used for describing disability in the normal US population [31] and to report incidence of disability [57], we acknowledge that disability is well-established as a continuum [58]. Particularly, our definition of incidence of disability neither provides information on the importance of the observed change nor considers relevant disability changes along all the possible SF-12 scores. One possible measure that might have been used is the decrease by more than one Standard Deviation (SD) of SF-12v2 scores from Wave 1 to Wave 2. However, the 25th percentile-based definition has been driven by the following reasons: firstly, to define persistence and incidence of disability likewise throughout the whole study; secondly, some SF-12v2 norm-based scores displayed only five possible values in our sample. Consequently, the use of a SD changes-based measure was not suitable for variables including limited number of values; thirdly, distribution change-based criteria are linked to the assumption that measurement error is constant across the range of possible scores. However, one study has reported that smaller SD are usually given at both scoring extremes [59]. Finally, whereas one amount of SD change may be perceived as highly important for some functioning domains along the disability continuum, the same amount can be perceived as less relevant in others. Nevertheless, to verify whether the use of this cut off-point (25th percentile) might have altered our results, two sensitivity analyses were conducted considering the 20th percentile and the 30th percentile in the SF-12 domains as other possible cut-off points for disability. The results obtained using these cut-off points were similar to those reported in the present paper (these data are available upon request). Another important limitation was that prevalence of incidence of disability was generally low. These small sample sizes had an impact on the statistical power of our analyses, specifically in the group of chronic MD (less than 100 observations). Hence, the lack of significant findings in the analyses of incidence should be interpreted with caution. Finally, although the measures of severity of symptoms for AUD and MD have been used previously, counting the number of symptoms is an indirect approach to assess severity, and it might have nothing to do with the clinical severity of symptoms [60].

## Conclusions

The present study has shown that chronic MD is an important risk factor for persistence and incidence of disability in the US general population. Chronic AUD were also predictors of persistence and incidence of disability for some areas of functioning. On the other hand, help-seeking was associated with persistent disability in chronic MD but not in the chronic AUD group. More effective actions might be necessary to facilitate help-seeking among persons with AUD. In addition, baseline help-seeking behavior did not prevent incidence of disability in chronic MD or AUD groups, and was even associated with incidence of disability in the mental health domain for chronic MD group. Further studies are needed to confirm this finding, adding to the model variables related to treatment quality and treatment compliance. Finally the present study has also confirmed that disability is not equally distributed in the population, since people with lower resources and suffering from comorbid health conditions might be more vulnerable to incidence and persistence of disability. Policymakers should realize that most of the budget cuts for mental health lead to negative consequences and higher long-term disability.

## References

[1] Hardeveld F, Spijker J, de Graaf R, Nolen WA, Beekman AT (2010). Prevalence and predictors of recurrence of major depressive disorder in the adult population. Acta Psychiatr Scand.

[2] Dawson DA, Goldstein RB, Grant BF (2007). Rates and correlates of relapse among individuals in remission from DSM-IV alcohol dependence: a 3-year follow-up. Alcohol Clin Exp Res.

[3] McCullough JP, Klein DN, Borian FE, Howland RH, Riso LP, Keller MB, Banks PL (2003). Group comparisons of DSM-IV subtypes of chronic depression: validity of the distinctions, part 2. J Abnorm Psychol.

[4] Miller PM: **The Nature of Addiction.** In *Principles of Addiction: Comprehensive Addictive Behaviors and Disorders.* Edited by Miller P. Academic Press 2013.

[5] Rubio JM, Markowitz JC, Alegria A, Perez-Fuentes G, Liu SM, Lin KH, Blanco C (2011). Epidemiology of chronic and nonchronic major depressive disorder: results from the national epidemiologic survey on alcohol and related conditions. Depress Anxiety.

[6] Atkinson C, Lozano R, Naghavi M, Vos T, Whiteford H, Murray C (2013). The burden of mental disorders in the USA: new tools for comparative analysis of health outcomes between countries. Lancet.

[7] Wittchen HU, Jacobi F, Rehm J, Gustavsson A, Svensson M, Jonsson B, Olesen J, Allgulander C, Alonso J, Faravelli C, Fratiglioni L, Jennum P, Lieb R, Maercker A, van Os J, Preisig M, Salvador-Carulla L, Simon R, Steinhausen HC (2011). The size and burden of mental disorders and other disorders of the brain in Europe 2010. Eur Neuropsychopharmacol.

[8] Zimmerman M, McGlinchey JB, Posternak MA, Friedman M, Boerescu D, Attiullah N (2006). Discordance between self-reported symptom severity and psychosocial functioning ratings in depressed outpatients: implications for how remission from depression should be defined. Psychiatry Res.

[9] Vittengl JR, Clark LA, Jarrett RB (2009). Deterioration in psychosocial functioning predicts relapse/recurrence after cognitive therapy for depression. J Affect Disord.

[10] Foster JH, Marshall EJ, Peters TJ (2006). Predictors of relapse to heavy drinking in alcohol dependent subjects following alcohol detoxification—the role of quality of life measures, ethnicity, social class, cigarette and drug use. Addict Biol.

[11] Dawson DA, Li TK, Chou SP, Grant BF (2009). Transitions in and out of alcohol use disorders: their associations with conditional changes in quality of life over a 3-year follow-up interval. Alcohol Alcohol.

[12] Ormel J, Oldehinkel AJ, Nolen WA, Vollebergh W (2004). Psychosocial disability before, during, and after a major depressive episode: a 3-wave population-based study of state, scar, and trait effects. Arch Gen Psychiatry.

[13] Rubio JM, Olfson M, Perez-Fuentes G, Garcia-Toro M, Wang S, Blanco C (2014). Effect of first episode axis I disorders on quality of life. J Nerv Ment Dis.

[14] Rubio JM, Olfson M, Villegas L, Perez-Fuentes G, Wang S, Blanco C (2013). Quality of life following remission of mental disorders: findings from the National Epidemiologic Survey on Alcohol and Related Conditions. J Clin Psychiatry.

[15] Lin EH, VonKorff M, Russo J, Katon W, Simon GE, Unutzer J, Bush T, Walker E, Ludman E (2000). Can depression treatment in primary care reduce disability? A stepped care approach. Arch Fam Med.

[16] Reed C, Monz BU, Perahia DG, Gandhi P, Bauer M, Dantchev N, Demyttenaere K, Garcia-Cebrian A, Grassi L, Quail D, Tylee A, Montejo AL (2009). Quality of life outcomes among patients with depression after 6 months of starting treatment: results from FINDER. J Affect Disord.

[17] Coulehan JL, Schulberg HC, Block MR, Madonia MJ, Rodriguez E (1997). Treating depressed primary care patients improves their physical, mental, and social functioning. Arch Intern Med.

[18] Feeney GF, Connor JP, Young RM, Tucker J, McPherson A (2004). Alcohol dependence: the impact of cognitive behaviour therapy with or without naltrexone on subjective health status. Aust N Z J Psychiatry.

[19] Hays RD, Wells KB, Sherbourne CD, Rogers W, Spritzer K (1995). Functioning and well-being outcomes of patients with depression compared with chronic general medical illnesses. Arch Gen Psychiatry.

[20] Rytsala HJ, Melartin TK, Leskela US, Lestela-Mielonen PS, Sokero TP, Isometsa ET (2006). Determinants of functional disability and social adjustment in major depressive disorder: a prospective study. J Nerv Ment Dis.

[21] Spijker J, Graaf R, Bijl RV, Beekman AT, Ormel J, Nolen WA (2004). Functional disability and depression in the general population. Results from the Netherlands Mental Health Survey and Incidence Study (NEMESIS). Acta Psychiatr Scand.

[22] Mojtabai R, Olfson M, Mechanic D (2002). Perceived need and help-seeking in adults with mood, anxiety, or substance use disorders. Arch Gen Psychiatry.

[23] Dawson DA, Grant BF, Stinson FS, Chou PS (2006). Estimating the effect of help-seeking on achieving recovery from alcohol dependence. Addiction.

[24] Spijker J: **Chronic Depression: Determinants and consequences of chronic major depression in the general population.*** PhD thesis*. University of Utrecht; 2002 [http://dspace.library.uu.nl/handle/1874/1525]

[25] Grant BF, Kaplan KD, Shepard J, Moore T (2003). Source and Accuracy Statement for Wave 1 of the 2001–2002 National Epidemiologic Survey on Alcohol and Related Conditions (NESARC).

[26] Grant BF, Hasin DS (1992). The Alcohol Use Disorder and Associated Disabilities Interview Schedule (AUDADIS).

[27] Chatterji S, Saunders JB, Vrasti R, Grant BF, Hasin D, Mager D (1997). Reliability of the alcohol and drug modules of the Alcohol Use Disorder and Associated Disabilities Interview Schedule–Alcohol/Drug-Revised (AUDADIS-ADR): an international comparison. Drug Alcohol Depend.

[28] Grant BF, Dawson DA (2006). Introduction to the National Epidemiologic Survey on Alcohol and Related Conditions. Alcohol Res Health.

[29] Ware J, Kosinski M, Keller SD (1996). A 12-Item Short-Form Health Survey: construction of scales and preliminary tests of reliability and validity. Med Care.

[30] Gandek B, Ware JE, Aaronson NK, Apolone G, Bjorner JB, Brazier JE, Bullinger M, Kaasa S, Leplege A, Prieto L, Sullivan M (1998). Cross-validation of item selection and scoring for the SF-12 Health Survey in nine countries: results from the IQOLA Project. Int Quality Life Assess J Clin Epidemiol.

[31] Rose MS, Koshman ML, Spreng S, Sheldon R (1999). Statistical issues encountered in the comparison of health-related quality of life in diseased patients to published general population norms: problems and solutions. J Clin Epidemiol.

[32] Koukouli S, Vlachonikolis IG, Philalithis A (2002). Socio-demographic factors and self-reported functional status: the significance of social support. BMC Health Serv Res.

[33] Scott KM, Von KM, Alonso J, Angermeyer MC, Bromet E, Fayyad J, de Girolamo G, Demyttenaere K, Gasquet I, Gureje O, Haro JM, He Y, Kessler RC, Levinson D, Medina Mora ME, Oakley Browne M, Ormel J, Posada-Villa J, Watanabe M, Williams D (2009). Mental-physical co-morbidity and its relationship with disability: results from the World Mental Health Surveys. Psychol Med.

[34] World Health Organization (1993). The ICD-10 Classification of Mental and Behavioural Disorders: Diagnostic Criteria for Research (ICD-10).

[35] Moreno C, Hasin DS, Arango C, Oquendo MA, Vieta E, Liu S, Grant BF, Blanco C (2012). Depression in bipolar disorder versus major depressive disorder: results from the National Epidemiologic Survey on Alcohol and Related Conditions. Bipolar Disord.

[36] Boschloo L, van den Brink W, Penninx BW, Wall MM, Hasin DS (2012). Alcohol-use disorder severity predicts first-incidence of depressive disorders. Psychol Med.

[37] Dawson DA, Saha TD, Grant BF (2010). A multidimensional assessment of the validity and utility of alcohol use disorder severity as determined by item response theory models. Drug Alcohol Depend.

[38] Wolter KM (1985). Introduction to Variance Estimation.

[39] Hardin JW, Hilbe JM (2003). Generalized estimating equations.

[40] StataCorp (2010). Stata Statistical Software: Release 11.

[41] Judd LL, Akiskal HS, Zeller PJ, Paulus M, Leon AC, Maser JD, Endicott J, Coryell W, Kunovac JL, Mueller TI, Rice JP, Keller MB (2000). Psychosocial disability during the long-term course of unipolar major depressive disorder. Arch Gen Psychiatry.

[42] Rhebergen D, Beekman AT, de Graaf R, Nolen WA, Spijker J, Hoogendijk WJ, Penninx BW (2010). Trajectories of recovery of social and physical functioning in major depression, dysthymic disorder and double depression: a 3-year follow-up. J Affect Disord.

[43] Blier P, Keller MB, Pollack MH, Thase ME, Zajecka JM, Dunner DL (2007). Preventing recurrent depression: long-term treatment for major depressive disorder. J Clin Psychiatry.

[44] Kruijshaar ME, Hoeymans N, Bijl RV, Spijker J, Essink-Bot ML (2003). Levels of disability in major depression: findings from the Netherlands Mental Health Survey and Incidence Study (NEMESIS). J Affect Disord.

[45] Volk RJ, Cantor SB, Steinbauer JR, Cass AR (1997). Alcohol use disorders, consumption patterns, and health-related quality of life of primary care patients. Alcohol Clin Exp Res.

[46] Daeppen JB, Krieg MA, Burnand B, Yersin B (1998). MOS-SF-36 in evaluating health-related quality of life in alcohol-dependent patients. Am J Drug Alcohol Abuse.

[47] Struening EL, Padgett DK (1990). Physical health status, substance use and abuse, and mental disorders among homeless adults. J Soc Issues.

[48] Goodell S, Druss BG, Walker ER, MAT MPH (2011). Mental disorders and medical comorbidity, vol 2.

[49] McMahon EM, Buszewicz M, Griffin M, Beecham J, Bonin EM, Rost F, Walters K, King M (2012). Chronic and recurrent depression in primary care: socio-demographic features, morbidity, and costs. Int J Family Med.

[50] Oleski J: **Predicting the new onset of perceived need for care and help-seeking for alcohol use disorders in the National Epidemiologic Survey on Alcohol and Related Conditions.** In *PhD Thesis*: University of Manitoba; 2011. [http://hdl.handle.net/1993/4960]

[51] Tucker JA, Simpson CA (2011). The recovery spectrum: from self-change to seeking treatment. Alcohol Res Health.

[52] Idler EL, Kasl SV (1995). Self-ratings of health: do they also predict change in functional ability?. J Gerontol B Psychol Sci Soc Sci.

[53] Clarke N, Mun EY, Kelly S, White HR, Lynch K (2013). Treatment outcomes of a combined cognitive behavior therapy and pharmacotherapy for a sample of women with and without substance abuse histories on an acute psychiatric unit: do therapeutic alliance and motivation matter?. Am J Addict.

[54] Bell NF, Blanchflower DG (2011). Youth unemployment in Europe and the United States. Nordic Economic Policy Review.

[55] Burnett-Zeigler I, Ilgen MA, Bohnert K, Miller E, Islam K, Zivin K (2013). The Impact of Psychiatric Disorders on Employment: Results from a National Survey (NESARC). Community Ment Health J.

[56] Wahlbeck K, McDaid D (2012). Actions to alleviate the mental health impact of the economic crisis. World Psychiatry.

[57] Joffe H, Chang Y, Dhaliwal S, Hess R, Thurston R, Gold E, Matthews KA, Bromberger JT (2012). Lifetime history of depression and anxiety disorders as a predictor of quality of life in midlife women in the absence of current illness episodes. Arch Gen Psychiatry.

[58] World Health Organization (2001). The International Classification of Functioning, Disablity and Health (ICF).

[59] Crosby RD, Kolotkin RL, Williams GR (2003). Defining clinically meaningful change in health-related quality of life. J Clin Epidemiol.

[60] Lux V, Aggen SH, Kendler KS (2010). The DSM-IV definition of severity of major depression: inter-relationship and validity. Psychol Med.

